# Traditional healers’ role in the detection of active tuberculosis cases in a pastoralist community in Ethiopia: a pilot interventional study

**DOI:** 10.1186/s12889-019-7074-9

**Published:** 2019-06-10

**Authors:** Bezawit Temesgen Sima, Tefera Belachew, Gunnar Bjune, Fekadu Abebe

**Affiliations:** 1 Department of Health Education and Behavioural Science, Institute of Health, Jimma University, P.O. Box 378, Jimma, Ethiopia; 2 Department of Population and Family Health, Institute of Health Science, Jimma University, P.O.Box 378, Jimma, Ethiopia; 3 Department of Community Medicine and Global Health, Institute for Health and Society, Faculty of Medicine, University of Oslo, P.O. Box 1130, Blindern, 0318 Oslo, Norway; 4 Oslo, Norway

**Keywords:** Tuberculosis, Traditional healers, TB control, Pastoralists

## Abstract

**Background:**

Pastoralists rely on traditional healers (THs) for general health problems. However, some studies indicate that such practices result in delays in the diagnosis and treatment of tuberculosis (TB) cases. This study aims to assess the role of traditional healers in the detection and referral of active TB cases in a pastoralist community.

**Methods:**

We identified 22 traditional healers from 7 villages of *Kereyu* pastoralist community in the Fentale district in Ethiopia in January 2015. We trained these THs in identifying presumptive TB symptoms and early referral to the nearby healthcare facilities. The training was held during a 1 week period that included a visit to their villages and follow-up. A 1 day meeting was held with the traditional healers, the district TB care and prevention coordinator and health extension workers from the selected sub-district to discuss the referral link between THs and the nearby healthcare facilities. Health providers working at the TB units in the selected healthcare facilities were oriented about the training given and planned involvement of THs in referring presumptive TB case. In addition, documentation of the presumptive TB cases was discussed.

**Results:**

We succeeded in tracing and interviewing 8 of the 22 THs. The rest were on seasonal migration. According to the THs report for the 1 year period, these 8 THs had referred 24 TB suspects to the healthcare facilities. Sputum smear microscopy confirmed 13 of the 24 suspects as having TB cases. Among those confirmed, 10 completed treatment and three were on treatment. Five presumptive TB cases were confirmed non TB cases through further evaluation at the healthcare facilities and six of the presumptive TB cases were lost to follow up by the THs. Whereas, four of the presumptive TB cases were lost to follow up to the healthcare facility.

**Conclusions:**

Results of the present study indicate that THs can contribute to the detection of undiagnosed active TB cases in a pastoralist community, provided they are given appropriate training and support.

**Electronic supplementary material:**

The online version of this article (10.1186/s12889-019-7074-9) contains supplementary material, which is available to authorized users.

## Background

About one third of the world’s population is infected with *Mycobacterium tuberculosis*, the causative agent of vTB, resulting in more than 10 million new cases and 1.6 million deaths globally in 2017 [1] . Ethiopia is among the top 20 high TB burden countries in 2017 and one of the 14 countries with a high burden of TB, TB/HIV, and MDR/RR-TB according to the 2018 WHO global TB report [1].

Pastoralists account for about 12% of the population in Ethiopia and they are the communities most affected by TB [2]. They mostly depend on traditional healers (THs) for their general health and illnesses, including TB. This holds true in most sub-Saharan countries where about 85% of the population visit THs regularly; in Ethiopia, up to 80% of the population use THs due to the relatively low cost of traditional medicine, easy access, and cultural acceptability of THs compared to modern healthcare facilities [3, 4].

The use of THs is a special challenge for TB prevention and care in the pastoralist area resulting in prolonged diagnostic and treatment delay of active TB cases [5, 6]. Studies also showed the presence of strong link between visiting THs, diagnostic and treatment delay and death due to TB [7, 8].

Ethiopia has developed a community health program called the Health Extension Program (HEP) to address primary health service coverage to rural communities. Health extension workers (HEWs) are community health workers who, since 2004, have been trained by the Federal Ministry of Health (FMoH) in Ethiopia to implement the HEP [9]. HEWs’ training includes presumptive TB case identification and referral, tracing lost-to-follow-up patients. They also employ WHO’s directly observed treatment short course (DOTS) strategy to provide treatment and other health education [9].

However, the HEP’s implementation faced a challenge in pastoralist communities in that most HEWs are selected from urban areas. This puts their service in doubt to address the pastoralists’ primary health care demands, and there is high turnover due to cultural differences between the HEWs and pastoralists; there is also a lack of adequate incentives for HEWs to provide adequate service to pastoralists [10, 11]. Likewise, because of pastoralists’ dispersion in a large geographical area and frequent migration, the construction of health posts is a challenge, making it difficult to implement the HEP including TB prevention and care services. Moreover, the health facilities are designed for sedentary way of life and are, for the pastoralists, hard to reach [11].

At present, TB control depends on passive case detection and treatment based on WHO’s DOTS strategy. However, due to their mobile lifestyle, poor access to health, education and other services including TB prevention and care services, pastoralists are not benefiting from the DOTS program in Ethiopia [12, 13]. Reports point out that pastoralist communities and rural residents can benefit from integration of THs to TB prevention and care services due to frequent consultation of THs [12, 14]. In addition, it has been shown that THs can serve as a bridge between health services and rural communities, where access to healthcare is poor [15, 16].

There is also ample evidence showing the contribution of THs to TB management and prevention [7, 17]. However, to our knowledge, no effort has been made to involve THs in TB prevention and control in Ethiopia. Therefore, this study evaluated the role of THs in the detection and referral of active TB cases in a pastoralist community.

## Methods

### Study area

This study was conducted in *Fentale (Kereyu*) Woreda (Woreda is equivalent to district) which is located in the east Shoa zone of Oromia, the southern part of the northern rift valley of Ethiopia. There are 18 rural kebeles (the smallest unit of district) of which 15 are pastoralist kebeles in *Kereyu*. The selected kebeles for this study are *Kobo*, *Benti, Dhabiti* and *D/Hadu*, *Tututi, Ilalla*, Gelchafrom pastoralist villages.

There are four health centers in the *Fentale* district, one health post in each village and one health center in Metehara city which is under the city’s administration.. There is also Metehara hospital which mainly provides services to non-pastoralists and Metehara sugar factory staff members. Only these two health facilities in Metehara provide sputum microscopic TB diagnostic service in the area and pastoralists need to travel to Metehara to get tested for TB. Moreover, there is no transportation system that links Metehara to most of the pastoralists’ villages in the district. Pastoralists need to walk for hours or the whole day to get to Metehara. In some cases they need to wait for a week to get transportation to this city (personal observation).

Health centers in the district are staffed with nurses and health posts with HEWs. TB presumptive symptom identification, referral and TB treatment is provided at the health posts or health centers in the study villages.

Traditional healing practitioners are referred to as ‘Traditional healers’ in this study, based on the definition given by the Ethiopian health system [4, 18]. One type of the THs is Faith healers, (called Sheekota, in the local language), who are believed to have some spiritual endowment and they use prayers to heal those who they believe are suffering from ‘bad sprit’ (Personal communication with HEWs). The most known THs are herbalists, followed by bone settlers, and birth attendants, locally called *Qoricha Aaada, Dhidhibaa*, and deesisttu, respectively (Personal Observation).

Herbalists (called *Qoricha Aaada* locally) are most common and they use wild plants (roots and leaves of plants) to treat common health problems like intestinal parasites and snake bites etc. Bone settlers (called Dhidhibaa/Dhidibtuu locally) practice realigning fractured bones, tooth extraction and cutting out the uvula. There are also traditional birth attendants (called Deesisttu Aadaa locally) who assist a woman during childbirth. They are highly respected community leaders and their opinions are highly regarded (Personal Observation). However, there is no data about the number of THs in the district and there is no registration or licensing system for the THs.

### Study design and sampling

**Fig. 1:**
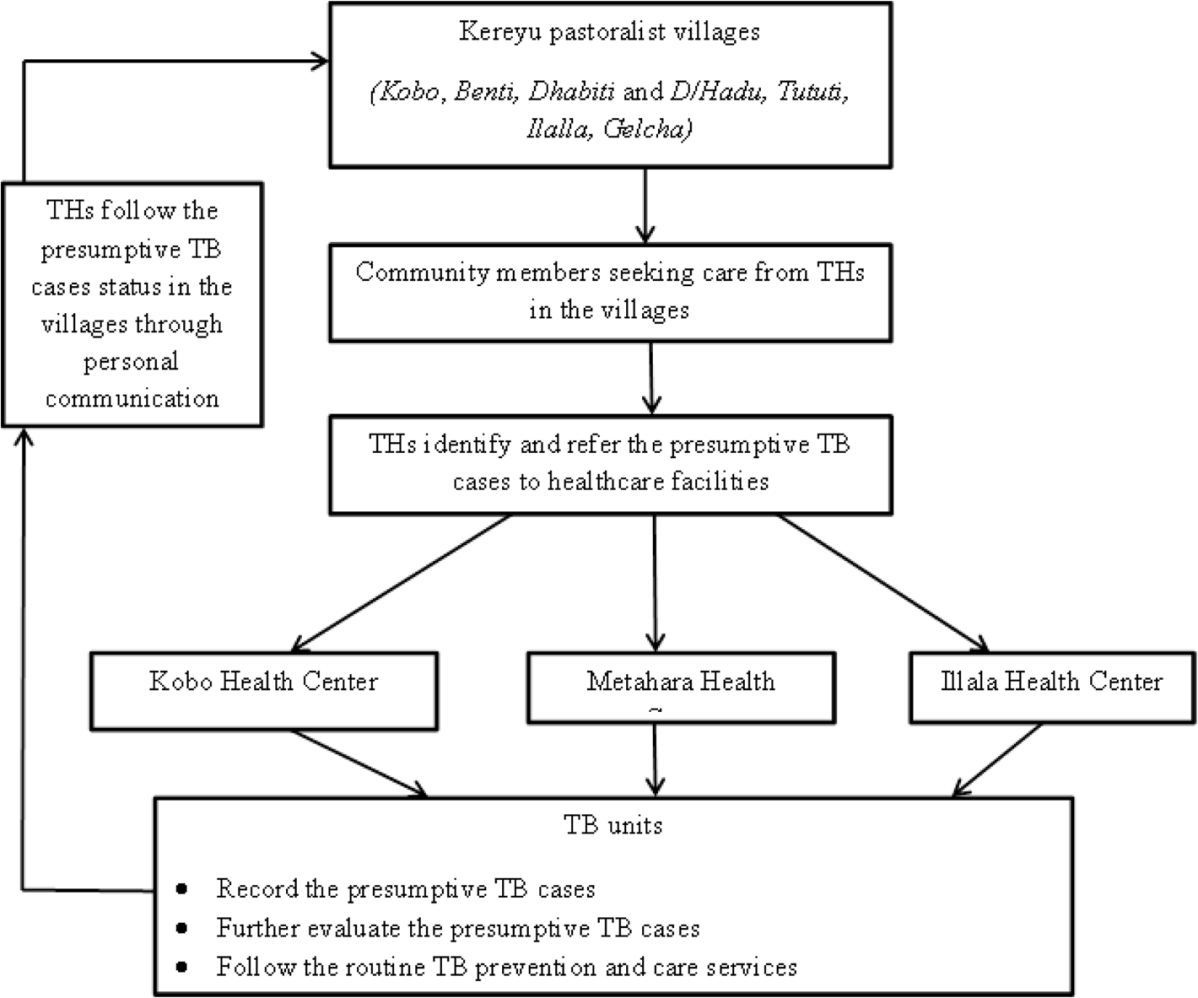
Diagrammatic presentation of the referral link between traditional healers and healthcare facilities and patient flow in this study

Seven pastoralist villages were selected from *Fentale* District in January 2015. The villages were selected conveniently, based on their population and availability of health center nearby. In these villages, we identified 22 THs who were well known and actively providing healing service to the community during the study period with the help of *kebele* leaders and HEWs. They were invited to participate in the study by HEWs who were employed at the health posts in the villages. Training was provided to the THs for a period of 1 week through oral communication and demonstration. Home visit and follow up were included. The THs were educated about symptoms suggestive of TB (coughing for more than 3 weeks, hemoptysis, weight loss, fever, or chest pain), the necessity and procedure of early referral to health care facility, the availability of free TB treatment and the importance of adherence to treatment to avoid drug resistance and early cure of the disease. Referral forms were prepared containing pre-filled information about the village, and indication that the person is a TB suspect and the need for further assessment at the health facility and the name and address of the TH referring the patient. The HEWs in the villages and the staff of the health facility’s TB unit were informed about the referral link with the THs. The person with presumptive symptoms of TB is sent to HEWs if available nearby or goes directly to the health facility for further management of the case. Pastoralists live in villages formed by related families in closely built houses. Since the THs are also pastoralists and live in the same villages with the community, the THs have access to information about the status of the presumptive TB cases after they have visited the health care facility. The district TB prevention and care coordinators, HEWs and THs were invited for a joint meeting to discuss the way forward. After 2 years of practicing presumptive TB case referrals, the THs were re-visited and interviewed about their knowledge regarding TB, the number of suspects they referred in the previous year, the status of those referred and whether they still refer suspects to health facilities. The referral link and the participant flow are presented in Fig. 1 below.

### Measurements

In August 2017, we collected the data about the THs socio demographic characteristics, knowledge regarding TB, their practice on presumptive TB case identification and referral using face to face interviews with semi-structured questionnaires, adopting from previous studies that were prepared in English [19, 20] and translated to Afaan Oromo (local language). In addition, we reviewed TB records from healthcare facilities linked with the THs through referral system (see Additional file 1).

### Knowledge about TB

The knowledge section had 14 questions on TB (about signs and symptoms indicating TB, transmission, cause and treatment of TB. The correct (yes) response to each question was given a score of “1” showing positive response and incorrect (no/I don’t know) response was given a score of “0” showing a negative response. The proportion of correct responses in each section were described and discussed.

### Presumptive TB case identification and referral practice of TH

Current presumptive TB case identification and referral practices for TB suspects were assessed for each TH, using 7 items. For each item, “yes” or “no” response was used and the proportion of correct (yes) and incorrect (no) responses corresponding to each item was reported, described and discussed.

### Record review at the respective healthcare facilities

We reviewed the healthcare facilities TB record book. We identified presumptive TB cases referred by THs and collected data on their presenting symptoms (cough for more than 2 weeks or more, productive cough, chest pain, weight loss, loss of appetite, fever, etc.). The presumptive TB cases diagnostic results (sputum smear microscopy) were reviewed and positive sputum smear microscopy was reported as confirmed TB cases and negative sputum smear microscopy and other diagnosis made by the health care providers (such as pneumonia, upper respiratory infection etc.) were reported as non TB cases. Data were also collected their treatment status. Treatment completed was reported for those who completed their treatment but did not confirm they are free from TB through sputum smear microscopy, cured (confirmed through sputum smear microscopy), on treatment (those who are still taking TB drugs).

### Data analysis

The data were entered and analyzed using the Statistical Software for Social Science (SPSS) version 22. We applied descriptive statistics to summarize the socio-demographic status, describe the knowledge and current practice of the THs on presumptive TB symptom identification and referral of suspect cases. Numbers and proportion of respondents in each section are reported. We were not able to run inferential statistics due to the small sample size. Due to seasonal migration and political instability in the region during the study period, we retrieved only 8 of the traditional healers.

## Results

Twenty-two THs were included in this pilot intervention study. However, only eight of them were available for interview after 2 years. The rest were unavailable due to seasonal migration and instability in the district during the study period.

Two of the eight THs were female and four were in the age range 31–60 years. All the respondents were from Oromo ethnic group and practice agro-pastoralism. Four of the THs could not read or write, and migrate seasonally.

Five out of eight THs mentioned bacteria as the cause of TB, while the rest mentioned living with active TB patients. All of them mentioned coughing for 2 weeks or more as the main symptom of TB followed by fever and night sweating (Table 1).

**Table 1:** Knowledge regarding TB among trained THs of Kereyu pastoralist, Ethiopia

Variables	Frequency (*n* = 8)
Heard of TB	8
Sources of TB knowledge	
Friends/family	3
Health workers	5
Cause of TB	
Bacteria/Germ	5
Living with untreated TB patient	3
Symptoms of TB	
Cough > 3 weeks	8
Coughing up blood	3
Weight loss	6
Shortness of breath	3
Fever and night sweating	5
Chest pain	2
Weakness/loss of appetite	6
Know TB is transmissible	6
Droplets from coughing and sneezing of a person with TB	7
Exposure to cold air	3
Sharing cups	4
TB can be prevented	8
Who could be infected by TB	
Anybody	5
Poor people	2
Homeless people	1
TB can be cured	8
Only specific drugs given by HCF can cure TB	8
Drugs are available for free	8

The THs mentioned that they referred 24 presumptive TB cases during a one-year period. Of these presumptive TB cases, 13 were confirmed to be TB and ten of them completed treatments while, three were still on treatment. Six of the presumptive TB cases were lost to follow up by the THs. The rest, five presumptive TB cases were non TB cases (Table 2).

**Table 2:** THs’ Presumptive TB case referral practice, Kereyu pastoralist, Ethiopia

Variables	Frequency	
	Yes	No
Ever seen a person with symptoms of TB	5	3
Ever referred TB suspect to HCF	5	3
Total number referred	24	
Have feedback on the progress of the patients	5	3
Willing to refer presumptive TB cases in the future	8	0

### Summary of data on referred presumptive TB cases from the healthcare facilities included in the study

A total of 20 TB suspects were recorded as referred by THs, and visited the respective healthcare facility linked by referral system with THs over a one-year period. The age of the TB suspects were from 14 to 75 with median of 31.5 (interquartile range = 32). Twelve of the presumptive TB cases were male. The presenting symptoms were coughing among five suspects, coughing and weakness (five); coughing, chest pain and weakness (five); coughing, fever, chest pain and loss of appetite (two) coughing and loss of appetite (one) coughing and fever (one) and coughing and sweating (one). Twelve were confirmed TB cases by sputum smear microscopy and ten had completed treatment whereas, two were still on treatment. Four of the presumptive TB cases referred by the THs did not report to the healthcare facilities linked through referral system with the THs.

## Discussion

THs have been involved in TB care and prevention activities in different developing countries and have been said to play an important role in TB control efforts [7]. The THs’ role in the detection of undiagnosed active TB cases in the pastoralist community has been examined in this study. Our finding showed that the THs referred 24 presumptive TB cases to the nearest health facility in a one-year period, out of which 13 were confirmed to be TB cases and 10 completed treatment. Different approaches have been suggested to strengthen the effectiveness of TB control such as DOTS, patient motivation, involving private sectors as well as community based treatment for TB in developing settings [21–23] . In a pastoralist community, engaging THs in TB control might help to facilitate the detection of undiagnosed active TB cases and treatment. This in turn may result in reduction of diagnostic and treatmnt delays and the spread of the disease in the community. This has been supported by similar studies where THs involvment in TB control has been reported to be successful [19, 24].

In pastoralist communities, involving the THs in TB control could support the existing community-based HEWs activity which is challenged by the frequent movement of the pastoralists, a factor which the HEP does not take into consideration [10]. The patoralists communities’ preference and frequent visits to THs [5, 6] could be an opportunity to reach presumptive TB cases through THs and link them with the health posts and health centers through referal system. This could fill the gap in the pastoralist areas. On the other hand, the HEWs could play important role in following up the THs by providing continuous health education and motivation as well as follow up of the collaboration.

This is also in line with WHO’s Stop TB strategy, which emphasizes that need for regular support, motivation, instruction and supervision for a sucessful link between healthcare facilities and community volunteers [25]. In the present study, we realised challenges such as the THs’ mobile lifestyle, financial constraint, short period of follow up and lack of motivation among the healthcare providers on maintaining sustainability of the link between THs and healthcare facility.. Further studies to develop guidelines, motivate the THs and integrate their activities with the national TB control program might help to improve the effort.

Concerning their knowledge of TB, five of the eight THs mentioned bacteria as the cause of TB while the rest mentioned living with active TB patient as the causes of TB. Coughing for 2 weeks or more, fever and night sweating were among the most mentioned symptoms of TB. Droplets from coughing and sneezing of an active TB patient was mentioned as the route of TB transmission by all the respondents. Interestingly, all the THs knew that TB drugs are avilable for free at healthcare facilities. This is in line with other similar studies in the pastoralist community [26, 27]. These results indicate that THs can be trained and can contribute to the detection of undiagnosed active TB case in the community .

All the eight THs mentioned that they still refer TB suspects to healthcare facility. This might indicate that THs can play an important role in presumptive TB case identification and referral in the pastoralist community where there is poor healthcare-seeking behaviour and poor access to healthcare facilities. This was suported by similar studies which reported the critical role THs could play in TB control programs in other settings [19, 20, 24]. In addition, studies from Ethiopia and other settings reported on the importance of integrating THs to existing healthcare system to improve health service delivery in rural settings [7, 28].

The absence of supplementary qualitative data and tracing back only few of the THs and being able to interview only 8 of the 22 THs are among the limitations of this study. Political instability in the region during the study period also limited our ability to conduct qualitative study. In addition, the THs reported referring 24 TB suspects to healthcare facilities while, from the healthcare facilities’ registry, we found only 20 TB suspects linked with the THs. This might be because the 4 TB suspects followed their treatment in health facilities where the referral link was not initiated. All the same, this is the first pilot study that involved THs in the pastoralist community and can be used as a baseline for further strengthening of the link between THs and conventional health service in the detection of undiagnosed active TB cases. Since there was no feedback system to THs from healthcare facilities, the only way THs could follow the TB suspects was to hear back from the patients after they had visited healthcare facilities. The THs efforts to get back to the patient was crucial to keep track of the outcome. This needs to be considered in future plans in the collaboration process. We recommend a large scale cluster randomized study to validate the reliability of such intervention in the pastoralist area before scaling up.

## Conclusions

Results of the present study indicate that THs can be trained and contribute to the detection of undiagnosed active TB cases in the community. Moreover, there is a possibility of integrating THs to the existing TB control service in the pastoralist community, provided they are given appropriate training, continuous follow up and motivation. Developing policy, guidelines, referral links and follow up for the collaboration between THs and the conventional health system could strengthen and sustain the collaboration.

## Additional file

Additional file 1: Questionnaire on post interventional assessments of traditional healers’ knowledge regarding TB and Presumptive TB case referral practice. (PDF 169 kb)

## Data Availability

The datasets used and/or analysed during the current study are available from the corresponding author on reasonable request.

## Abbreviations

DOTS: Directly observed treatment short course
FMoH: Federal Minster of Health
HEWs: Health Extension Workers
NSD: The Norwegian Social Science Data Service
SPSS: Statistical Software for Social Science
TB: Tuberculosis
THs: Traditional healers
WHO: World health organization
