# Supplementary material for: Traditional healers’ role in the detection of active tuberculosis cases in a pastoralist community in Ethiopia: a pilot interventional study
Source: BMC Public Health. 2019 Jun 10;19:721. doi: 10.1186/s12889-019-7074-9 (PMC6558710; doi:10.1186/s12889-019-7074-9)
Supplement: Supplementary file 1 — Questionnaire on post interventional assessments of traditional healers’ knowledge regarding TB and Presumptive TB case referral practice. (PDF 169 kb) [file 12889_2019_7074_MOESM1_ESM.pdf]

Supplementary: Questionnaire on post interventional assessments of traditional healers' knowledge regarding TB and Presumptive TB case referral practice

Part I. Socio- demographic characteristics of the respondents

1. ID number of respondent_________________________________
2. District ____________________________Kebele__________________
3. Village________________________________ House No._____________
4. Sex : 1= Male 2= Female
5. Age (Year): ____________
6. Ethnicity: 1= Oromo 2= Amhara 3=other (specify) ________________________________
7. Religion 1= Muslim 2= Christianity 3= other specify _________________________________
8. Marital status 1= Married 2= single 3 = divorced 4= widowed
9. Educational status: 1= can't read & write 2= read & write 3= primary (1-8) 4= secondary (9-12) 6= other (specify)_______________________
10. Occupation: 1= Nomadic pastoralist 2= Agro-pastoralist 3= farmer 4= Business 5= Civil servant 6= student 7= other (specify) ____________________________
11. Do you migrate in dry Seasons?

    1= Yes 2= No

Part II. Assessments of knowledge about PTB

1. Have you ever heard about the disease called PTB?

   1= Yes 2= No

2. If yes, from whom mainly?

   1= from friend (s) 2= From health workers /Health extension workers

   3= From media (radio, TV poster etc) 4= from patient 5= family

3. member/friends 6= Other (specify): ___________________________

4. What do you think is the cause of PTB? (Check all that are mentioned)

   1= Bacteria/germ 2= witchcraft 3= shortage of food 4= unventilated house

   5= Sexual overindulgence 6= hard work 7= poverty 8= living together with untreated

   TB patient 9= Cold air 10= others (specify):______________________

5. What are some of the common symptoms of TB? (Check all that are mentioned)

   1= cough 2= Cough for 2 or more weeks 3= cough up blood 4= weight loss

   5= shortness of breath 6= Fever/sweat at night 6= chest pain 7= weakness or loss of

   appetite 8= rash 9= other (Specify)______________ 10= do not know

6. Do you know that TB can be transmitted?

   1= Yes 2= No

7. If yes to Q7, How can TB be transmitted from person to person ? (Check all that are mentioned)

   1= through droplets from coughing or sneezing of a person having TB

   2= hand shaking of a person having TB 3= kissing TB infected person

4= drinking raw milk 5= eating TB infected meat

6= exposure to cold air 7= sharing cup with a person having TB

8= sharing utensils 9= chewing chat together

10= Others (specify) :______________

8. Who do you think can transmit TB? (Check all that are mentioned)

1= a person who have active TB and not taking TB drugs

2= Anyone who has cough

3= a person who is diagnosed having TB and finished treatment

4= others specify

9. Do you think that the transmission of PTB can be prevented?

1= Yes 2= No

10. If Yes to Q12, How can TB be prevented (Check all that are mentioned)

1= using separate room 2= covering mouth and nose while coughing or sneezing

3= avoiding sharing cups 4= avoiding eating raw meat

5= avoiding drinking raw milk 6= through good nutrition

7= by prayer 8= others (specify):___________________

11. In your opinion, who can be infected with TB? (Check all that are mentioned)

1= anybody 2= Poor people 3= homeless people 4= only alcoholics

5= only people living with HIV 6= Others (specify): __________

12. Can TB be cured?

1= Yes 2= No

13. If Yes to Q15, How can someone with TB be cured? (Check all that are mentioned)

1= traditional Medicine 2= home rest without medicine 3=praying

4= specific drugs given by health facility 5= DOTS

6= do not Know 7= others (specify): ______________

14. Do you know that the drugs are available for free?

1= Yes 2= No 3= I don't know

Part III: presumptive TB case identification and referral practice of traditional healers

1. Have you ever seen a person with signs and symptoms of TB in the last one year?

   1=Yes 2=No

2. If yes, have you ever referred a person with symptoms of TB to healthcare facilities?

   1=Yes 2=No

3. If yes how many people with presumptive TB symptoms did you refer? Please specify:_____________________

4. Have you heard back from the patients you referred after their visit to health facilities?

1=Yes 2=No

5. If yes, what was their status after visiting healthcare facility?

   1= non TB cases: _______________
   2= sputum examined and confirmed TB cases: __________
   3= treatment completed: __________________
   4= on treatment: _______________
   5= lost to follow up: _______________
   6= other, please specify:_________________

6. Are you still referring presumptive TB cases to healthcare facilities?

   1=Yes 2=No

7. Are you willing to refer presumptive TB cases in the future?

   1= Yes 2=No

Record review form about the status of referred presumptive TB cases from healthcare facilities linked by referral system with the THs

1. **Health facility___________ 2. Village ______ 3. date of referral ____________**

**Sr. no**	**Age**	**Presenting symptom/s**	**TB diagnostic result (√)**		**Diagnosis (√)**		**Treatment outcome (√)**					
			**positive**	**Negative**	**TB**	**Others**	On treatment	Treatment completed	Treatment discontinued	**Cured**	**Died**	**Referred**
